# Alanine Aminotransferase as a Monitoring Biomarker in Children with Nonalcoholic Fatty Liver Disease: A Secondary Analysis Using TONIC Trial Data

**DOI:** 10.3390/children5060064

**Published:** 2018-05-25

**Authors:** Idil Arsik, Jennifer K. Frediani, Damon Frezza, Wen Chen, Turgay Ayer, Pinar Keskinocak, Ran Jin, Juna V. Konomi, Sarah E. Barlow, Stavra A. Xanthakos, Joel E. Lavine, Miriam B. Vos

**Affiliations:** 1 H. Milton Stewart School of Industrial & Systems Engineering, Georgia Institute of Technology, 755 Ferst Dr, Atlanta, GA 30318, USA; idilarsik@gatech.edu (I.A.); dfrezza3@gmail.com (D.F.); wchen424@gatech.edu (W.C.); ayer@isye.gatech.edu (T.A.); pinar@isye.gatech.edu (P.K.); 2 Department of Pediatrics, Emory University School of Medicine, 1760 Haygood Dr, Atlanta, GA 30322, USA; jfredia@emory.edu (J.K.F.); jinr@usc.edu (R.J.); junakonomi@emory.edu (J.V.K.); 3 Department of Pediatrics, University of Texas Southwestern, 5323 Harry Hines Blvd, Dallas, TX 75390, USA; sarah.barlow@utsouthwestern.edu; 4 Department of Pediatrics, University of Cincinnati College of Medicine, Cincinnati Children’s Hospital, 3333 Burnet Ave, Cincinnati, OH 45229, USA; stavra.xanthakos@cchmc.org; 5 Department of Pediatrics, Columbia University, 3959 Broadway, New York, NY 10032, USA; jl3553@columbia.edu

**Keywords:** NASH, ALT, pediatrics, fibrosis

## Abstract

Background: Validated noninvasive biomarkers to assess treatment response in pediatric nonalcoholic fatty liver disease (NAFLD) are lacking. We aimed to validate alanine aminotransferase (ALT), a monitoring biomarker for change in liver histology. Methods: A retrospective analysis using data from the TONIC trial. NAFLD histologic assessments were defined by: Fibrosis score, NAFLD activity score (NAS), nonalcoholic steatohepatitis (NASH), and a combination of NASH resolution and fibrosis (NASH + fibrosis). Analysis was performed using classification and regression trees (CART) as well as logistic regression. Results: Mean ALT for the child over 96 weeks and percent change of ALT from baseline to 96 weeks were significant predictors of progression of NAFLD for each histologic assessment (*p* < 0.001 for fibrosis score, NASH, and NASH + fibrosis and *p* < 0.05 for NAS). Mean ALT adjusted for age, sex and ethnicity was a better predictor for change in NASH (81.8 (11.0) ROC (receiver operating characteristic curve) mean (SD (Standard derivation))) and NASH + fibrosis (77.8 (11.2)), compared to change in NAS (63 (17.7)) and fibrosis (58.6 (11.1)). Conclusion: Mean ALT over 96 weeks is a reasonable proxy of histologic improvement of NASH and NASH + fibrosis. These findings support ALT as a valid monitoring biomarker of histologic change over time in children with NASH and fibrosis.

## 1. Introduction

There is an urgent need for effective therapies for nonalcoholic fatty liver disease (NAFLD) because of its high prevalence, affecting approximately 25% of the global population [1,2]. It is currently the second leading indication for liver transplant in the US [3]. Children are frequently affected by NAFLD and the prevalence is increasing [1,4]. Because of this, clinical trials for potential therapies have been conducted in children with NAFLD [5,6] and new studies are needed to test novel therapeutic approaches [7].

NAFLD is a disease that progresses slowly and clinical outcomes such as cirrhosis, portal hypertension, and liver transplant develop potentially decades after the initial onset of the disease. Because of this, histology has become a surrogate marker of future outcomes based on limited data showing that fibrosis is related to mortality [8]. While liver biopsies are commonly performed and histology is recommended as the best primary outcome for phase 3 clinical trials in pediatric NAFLD [6,9], they are both invasive and costly [10,11] and biopsies are typically avoided in early phase studies.

Alanine aminotransferase (ALT) is a serum marker of liver damage; it is routinely measured in clinical labs and is reproducible and accurate. Intuitively, a large decrease or normalization of ALT may reflect decreased inflammation [12]. Hence, ALT is commonly used to monitor inflammatory liver diseases, e.g., autoimmune hepatitis, and liver transplantation for routine clinical monitoring. Most clinicians caring for NAFLD patients routinely obtain ALT to assess change over time; however, the literature has mixed support for this approach and it is uncertain whether ALT is valid for measuring change in inflammation and liver damage. A previous analysis of ALT in the clinical trial of treatment of nonalcoholic fatty liver disease in children (TONIC) showed that for every decrease of 10 U/L, the relative odds of histologic improvement and resolution of nonalcoholic steatohepatitis (NASH) were 1.31 and 1.26 respectively [13]. However, other studies have suggested that ALT is not associated with liver histology in NAFLD [14] and because of this, the utility of ALT as a surrogate marker of response to treatment in early phase NAFLD clinical trials continues to be questioned. Therefore, the purpose of this study was to further explore data from the TONIC trial using predictive modeling to test ALT as a predictor of histologic progression versus improvement of pediatric NAFLD over time.

## 2. Materials and Methods

Permission to conduct the analysis and de-identified data were obtained from the National Institute of Diabetes and Digestive and Kidney Diseases (NIDDK) Repository and approved by the Emory IRB. This was a secondary analysis using data from the 96 week, randomized, controlled TONIC trial comparing placebo to metformin or vitamin E, along with standard of care [5]. Inclusion criteria included persistently elevated ALT > 60 U/L and a biopsy-confirmed NAFLD. ALT was measured 10 times during the study, including study entry and at 96 weeks. Four different histologic assessments, that have been commonly used in NAFLD studies, were considered: (1) fibrosis stage [15], (2) NAFLD Activity Score (NAS) [16], (3) NASH [17] and (4) a combined outcome of NASH resolution and fibrosis score (NASH + fibrosis), [9] NASH resolution and fibrosis score was combined because these are both measurements of NAFLD severity. Each assessment was performed at baseline and at 96 weeks. Summarized definitions for each assessment in Table 1.

### 2.1. Fibrosis Assessment

**Figure 1:**
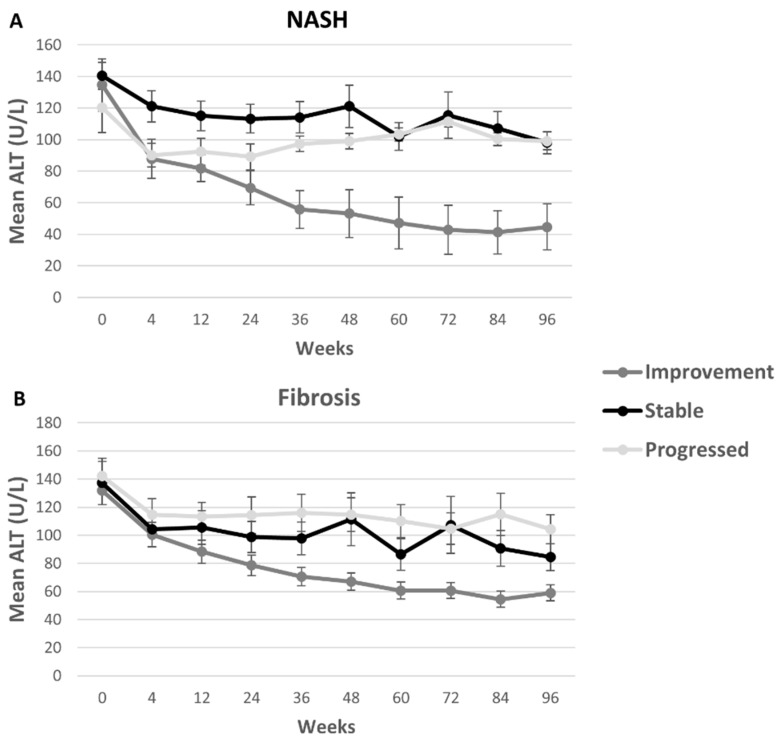
Mean ALT concentrations were higher among stable and progressing groups than among improving participants, when measured by the NASH assessment. Mean ALT concentrations had similar initial trajectories when measured by the fibrosis score, with the improvement group dropping approximately 45%. Mean ALT is shown at each time point for participants that showed progression (lightest gray), improvement (dark gray) and that stayed stable from baseline to 96 weeks (black) for NASH assessments (**A**) and for fibrosis (**B**). ALT: alanine aminotransferase; NASH: nonalcoholic steatohepatitis.

**Figure 2:**
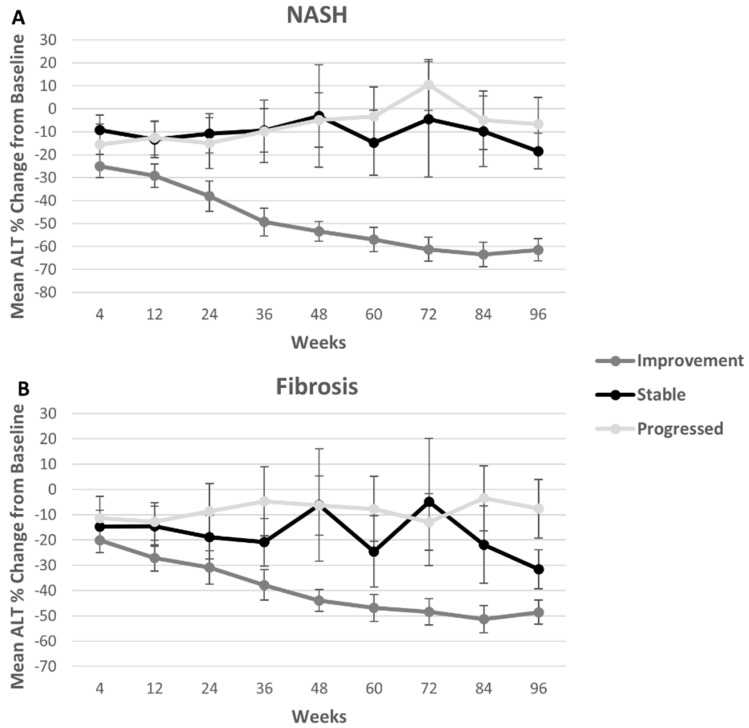
Changes in ALT concentrations from baseline were similar in stable patients compared to those with progressed disease when measured by the fibrosis and NASH assessment, though with the fibrosis score participants were more variable. The percent change in ALT from baseline is shown at each time point for participants that showed progression (lightest gray), improvement (dark gray) and that stayed stable from baseline to 96 weeks (black) was added for NASH assessments (**A**) and fibrosis (**B**). ALT (alanine aminotransferase); NASH (nonalcoholic steatohepatitis).

There are seven fibrosis stages (0 = None, 1a = Mild zone 3, 1b = Moderate zone 3, 1c = Portal/periportal, 2 = Zone 3 and periportal, 3 = Bridging, and 4 = Cirrhosis) [15]. The fibrosis scores of 1a, 1b and 1c were considered as a single score of 1. Improvement in fibrosis was defined as any decrease in stage and progression in fibrosis was defined as increase in numbered stage. Subjects that maintained their fibrosis score at 96 weeks compared to baseline were labeled stable. Subjects with fibrosis score of 0, meaning no fibrosis at baseline, and remaining fibrosis-free at 96 weeks were considered stable for Figure 1 and Figure 2 and excluded from regression models.

### 2.2. NAFLD Activity Score

NAFLD activity score (NAS) is a sum of steatosis (0–3), inflammation (0–3) and ballooning (0–2) scores. Improvement in NAS was defined as at least 2 point decrease in NAS [16] from 0 to 96 weeks. Progression was defined as any increase or decrease of 1 point. Stability was not defined for this assessment due to the lack of concrete clinical definition.

### 2.3. NASH

NASH was dichotomized, defining the score of 0 as no steatohepatitis (steatosis only) versus any steatohepatitis (score of 1), which included borderline steatohepatitis and definite steatohepatitis [17]. Improvement in NASH was defined as resolution of steatohepatitis from score 1 to 0 (no steatohepatitis) and progression in NASH were defined as change from no steatohepatitis (0) to any steatohepatitis (1). In Figure 1 and Figure 2, subjects with persistent NASH, not improving nor worsening, from baseline to 96 weeks were considered stable in NASH. Subjects with NASH assessment of 0 at screening and at 96 weeks were also considered stable for the figures and excluded from regression models evaluating NASH improvement.

### 2.4. NASH Resolution and Stable Fibrosis (NASH + Fibrosis)

This benchmark is a combination of NASH resolution (improvement) and decrease or no change in fibrosis score [18]. Progression was defined as no resolution and any increase or no change in fibrosis. Stability was not defined for this assessment due to the concrete clinical definition of NASH.

### 2.5. Data Preparation

The initial sample consisted of all patients in the TONIC trial with a biopsy at baseline and at 96 weeks (*n* = 173). ALT greater than 500 U/L (*n* = 3) or missing at either time point (*n* = 24) were excluded. Therefore, 146 patients were included in these analyses. Clinical characteristics and demographics for baseline and 96 are summarized in Table 2. For NASH + fibrosis, only patients with NASH at baseline (*n* = 119) were included.

### 2.6. Predictive Model Development

For the prediction of NAFLD improvement or progression, two variables were calculated, (1) mean ALT over the 96-week trial period (average of ALT at all 10 time points) and, (2) percent change of ALT at week 96 compared to baseline. Using the dynamic time warping distance [19], which is a technique for comparing time series providing the least cumulative distance between two time series, we measured ALT concentrations at baseline and at week 96. Hierarchical clustering was performed and patients were divided into 5 clusters. These were based on patients with (1) no change, (2) sharp increase, (3) slight increase, (4) sharp decrease, (5) slight decrease. The distance between two time series was determined using dynamic time warping (DTW). DTW finds the optimal alignment between two time series. The cluster information on ALT was included in the model building process. The patient demographics such as age, sex, and ethnicity were also considered as control variables.

Classification and Regression Trees (CART) and logistic regression were performed to predict the utility of ALT to determine progression or improvement. CART is a classification method which uses historical data to construct decision trees. The constructed tree can then be used for classification of new observations. The variables used in fully adjusted predictive models were age, gender, ethnicity, ALT cluster, ALT at baseline and at 96 weeks, % change in ALT from baseline, coefficient of variation of ALT, and mean ALT over the trial period (average of all time points) for each of the 4 histologic assessments. To account for the small sample size, *k*-fold cross validation was used to estimate the model performance. The data was divided into *k* = 5 subsamples of equal size. Each subsample was used once for testing while a model was trained on the remaining four subsamples. This procedure was repeated 20 times using different (randomly selected) 5 folds. The average model performance (area under the receiver operating characteristic (ROC) curve (AUC)) was calculated across 20 replications and the final model was fitted to the entire data.

ROC curve was used to measure the sensitivity and specificity of the candidate marker for the prediction of histologic improvement in NAFLD. Progression was considered the positive case while improvement was considered the negative case for the calculations. Univariate logistic regression models were used to identify the significance of mean ALT over two defined trial periods (48 vs. 96 weeks) and percent change of ALT compared to baseline at week 96. To assess the necessary length of ALT monitoring, the mean ALT was calculated based on 48 weeks of data and compared with the mean ALT over the 96-week trial period. All analyses were performed using R version 3.1.3.

## 3. Results

The original subject demographics were previously published [4]. For this analysis, demographics were grouped by the four NAFLD histologic assessments and progression or improvement for that histologic assessment (Table 2). Subjects were on average 13 years old [13.2 yrs (2.5)], 82% female, and 73% white and 64% Hispanic. The mean BMI z-score was 2.7 (0.7). Mean ALT was higher in the group that progressed versus the group that improved at each time point with the exception of NAS Score and NASH at baseline. For NASH and fibrosis score, those subjects that remained stable had mean ALTs between 150 U/L and 90 U/L over the course of the study (Figure 1). Percent change calculated from baseline was largest in the improvement group as expected. Changes in ALT concentrations from baseline were similar in stable patients compared to those with progressed disease when measured by the fibrosis and NASH assessment, though with the fibrosis score participants were more variable (Figure 2).

### Associations between Mean ALT and NAFLD

The univariate logistic regression indicated that mean ALT over 96 weeks was a significant predictor of progression of NAFLD for each histologic assessment (*p* < 0.001 for fibrosis score, NASH, and NASH + fibrosis and *p* < 0.05 for NAS). Similarly, the univariate logistic regression indicated that percent change in ALT (at week 96 compared to baseline) was also a significant predictor of progression of NAFLD for each histologic assessment (*p* < 0.001 for NAS, NASH, and NASH + fibrosis and *p* < 0.01 for fibrosis score). For one unit of increase in the mean ALT, the odds of progression increased by a factor of 1.02, 1.01, 1.04, and 1.05 for fibrosis score, NAS, NASH, and NASH + fibrosis respectively. Similarly, for 1% increase in ALT change, the odds of progression increased by a factor of 1.01, 1.03, 1.04, and 1.03 for fibrosis score, NAS, NASH, and NASH + fibrosis respectively. The mean ALT over 48 weeks was statistically significant for progression of NAFLD for all scores except for NAS (*p* < 0.01; data not shown). The average model performances (AUC, Sensitivity, Specificity) using a univariate logistic regression model with mean ALT over 96 weeks showed better performance for NASH (85.6 (9.3); 71.1 (15.7); 84.4 (10.7)) and NASH + fibrosis assessments (84.9 (8.6); 67.9 (16.8); 85.7 (11.2)) than fibrosis (69.6 (10.4); 62.9 (12.5); 70.9 (14.6)) and NAS (65.1 (10.7); 57.9 (12.4); 64.6 (16.4)).

Using the CART method for the fully adjusted model, the threshold for mean ALT to signify improvement using the fibrosis score was 77.1 U/L (Table 3). For NASH, the threshold for ALT at 96 weeks (the time of second biopsy) to signify improvement was 56.5 U/L. For NAS, the threshold for percent change in ALT between baseline and 96 weeks to signify improvement was 68% (Table 3). Using only mean ALT over 96 weeks (Table 3) demonstrated a narrow range of mean ALT to predict improvement (62–77 U/L).

The average model performances (AUC, Sensitivity, and Specificity) of these classification trees for each histologic assessment and each model show better specificity and sensitivity for NASH and NASH + fibrosis assessments (Table 4). The models with mean ALT at 48 weeks had lower specificity and sensitivity compared to those with mean ALT at 96 weeks and percent change in ALT (data not shown). Overall, the models for NASH and NASH + fibrosis performed better than NAS and fibrosis score.

## 4. Discussion

Understanding the strengths and weaknesses of ALT as a biomarker of histologic change is valuable for clinical care and early phase clinical trials in pediatric NAFLD in which ALT is utilized as an outcome in future. There are several approaches to determining validity of a biomarker, including to assess the truthfulness of a measure, the discriminant validity, and the feasibility [20]. According to the BEST (Biomarkers, Endpoints, and other Tools) Resource, the definition of clinical validation is “Establishing that the test, tool, or instrument acceptably identifies, measures, or predicts the concept of interest [21]”. ALT has long been accepted as a truthful measure of liver inflammation and ALT is highly feasible because it is widely available, requires only a small amount of blood and is relatively inexpensive. However, the sensitivity to change (discriminant validity) is not well documented in pediatric NAFLD. In this analysis, we found that the mean ALT for a child with NAFLD over 96 weeks discriminated well between those who progressed as defined by all four histologic assessments we tested (fibrosis, NAS, NASH, and NASH + fibrosis) compared to improvement. Specifically, a mean ALT over 2 years of less than 62 (for NASH) and 77 U/L (for fibrosis) was strongly associated with improvement in histology.

We tested several ways to quantify ALT changes including percent change, mean ALT over 48 weeks, and mean change over 96 weeks using predictive modeling. These comparisons have led to several important observations. First, we found that mean ALT, which may be easier to utilize in clinical setting, performed better than percent change in ALT. Second, our analysis has shown that, mean ALT over 96 weeks was more sensitive and specific than mean ALT over 48 weeks for all four histologic assessments. This may be due in part because the second histologic assessment was at 96 weeks and thus more of the measurements were closer in time to the liver biopsy with the 96-week mean ALT. Overall, CART with mean ALT, which is easy to implement in clinical practice, produced strong predictive models for NASH and NASH + fibrosis.

ALT has been commonly used in early stage clinical trials for NAFLD and is used frequently in the clinical setting. Our study adds to this by assessing the “mean” ALT, rather than a single time-point ALT. Mean ALT proved to have greater association with histologic change compared to single time point, particularly when compared to the final measurement of this clinical trial. Mean ALT is a significantly less invasive assessment than liver biopsy for detecting progression of NAFLD in the clinical setting and so it is valuable to understand how predictive it is. While liver biopsy is commonly used as a gold standard, it has limitations. Furthermore, liver biopsies are subject to intra- and inter-observer variability in the grading of steatosis, fibrosis staging, and NASH diagnosis [22]. Finally, liver biopsies have a small but important risk of complications [23].

According to recommendations, biomarkers proposed for use in disease monitoring should not only be more convenient but also reflect a biologic activity in the disease process. Importantly, ALT meets this criteria. However, ALT as a biomarker has limitations, particularly when used at a single time point. For example, a cross sectional pediatric NAFLD study demonstrated a wide range of severity of disease in children by liver biopsy despite normal ALT levels [14]. Our findings suggest that ALT over time (mean ALT) is more useful because it can reflect improvement or progression, rather than a specific stage of disease. Of note, applying our findings would require having a baseline liver biopsy with an ALT near in time and subsequently measuring the ALT repeatedly over time. Further, this study included only children with ALT > 60 at baseline so it is unclear what mean ALT and change of ALT over time means for children with lower ALT at the time of liver biopsy.

Importantly, in this study, we quantified the longitudinal association of ALT to histologic change and not simply a cross-sectional association. Verma et al. examined the cross-sectional relationship between ALT and both the NASH and fibrosis histologic assessments in adults [24]. They reported that a single ALT level is not a good predictor for NASH or fibrosis. We agree that a single ALT measure will not accurately predict NASH or fibrosis, but rather we show that in patients with both histologic disease and an elevated ALT > 60, the change in mean ALT over time is a reasonable biomarker of improved histology.

There were some limitations to this study. For therapeutic development, clinically meaningful endpoints directly measure how a patient feels, functions or survives. Liver histology is itself a surrogate outcome because it is not a direct measure of how a patient feels, functions or survives. Hence, comparison of ALT to liver histology is less meaningful, as it is a “surrogate of a surrogate”. To better validate ALT, a future comparison of mean ALT to long-term (10 and 15 year) clinical outcomes is needed. Due to the small data set, *k*-fold cross validation was used. A larger data set would allow a hold-out method of validation, which is superior to k-fold cross validation. Some patients had missing values on ALT measurements. Although the number of missing ALT measurements was small, this might have affected the mean ALT levels over 48 and 96 weeks. All of the children in this study had elevated ALT at baseline (ALT > 60 U/L) and we were unable to study ALT as a predictor of outcomes of NAFLD in children with lower ranges of ALT. Studies have shown that NAFLD, including NASH with fibrosis, can exist even in children with normal to mildly elevated ALT, though severe disease is more likely to be present at higher ranges of ALT [14]. Further, as evidenced by the CyNCh trial, ALT can improve without improvement in histology. The application of ALT as a surrogate marker, therefore, needs to be further defined and validation in other clinical trials is recommended to establish generalizability of these findings.

Strengths of the analysis include the use of high quality data collected in a clinical trial including rigorous phenotyping of liver histology at baseline and end of treatment. Most participants had >95% of ALT measurements available and all had two liver biopsies, two years apart. ALT as a biomarker is cheap, commonly available and uniform across various labs [25].

## 5. Conclusions

In summary, this analysis is the first to study and confirm the use of mean ALT averaged over time as a biomarker of histologic improvement or progression in children with elevated ALT and biopsy-confirmed NAFLD. In children with an ALT > 60 at baseline, a mean ALT at or less than 62-77 U/L over time predicted improvement in NASH and fibrosis.

## Figures and Tables

**Table 1:** Histologic assessment definitions.

	Improvement	Progression	Stable
Fibrosis	Any decrease in stage	Any increase in stage	No change in fibrosis score when comparing baseline to 96 weeks
NAS	At least two-point decrease in NAS	Any increase or decrease by 1 in NAS	N/A
NASH	Resolution of steatohepatitis or borderline NASH to none	Change from none to NASH or borderline NASH	No change or change between NASH and borderline NASH when comparing baseline to 96 weeks
NASH + Fibrosis	NASH score of 0 and a decrease or no change in fibrosis	NASH score of 1 or higher and increase or no change in fibrosis	N/A

NALFD: nonalcoholic fatty liver disease; NAS: NAFLD Activity Score; NASH: nonalcoholic steatohepatitis.

**Table 2:** Mean baseline demographics.

	Fibrosis		NAS		NASH		NASH + Fibrosis	
	Improve (*n* = 57)	Progress (*n* = 71)	Improve (*n* = 68)	Progress (*n* = 77)	Improve (*n* = 50)	Progress (*n* = 81)	Improve (*n* = 39)	Progress (*n* = 73)
**Age, year;** **(mean (SD))**	13 (2.2)	13 (3)	13 (2)	13 (3)	12.9 (±2.0)	13 (3)	12.6 (1.7)	13 (3)
**Female (*n* (%))**	46 (81%)	60 (85%)	56 (82%)	62 (81%)	42 (84%)	66 (82%)	34 (87%)	59 (81%)
**Ethnicity (*n* (% Hispanic))**	36 (63%)	46 (65%)	46 (68%)	47 (61%)	34 (68%)	49 (61%)	26 (67%)	47 (64%)
**BMI z-score (mean(SD))**	2.7 (0.6)	2.7 (0.7)	2.6 (0.7)	2.8 (0.6)	2.7 (0.7)	2.8 (0.7)	2.6 (0.7)	2.7 (0.6)
**ALT (U/L; mean(SD))**	132 (75)	138 (70)	144 (84)	122 (62)	126 (72)	139 (71)	128 (79)	147 (71)
**Pre-Pubertal (*n* (%))**	25 (44%)	39 (55%)	14 (21%)	58 (75%)	17 (34%)	48 (59%)	14 (36%)	38 (52%)
**Fibrosis (*n* (%))**								
**0**	0 (0%)	18 (25%)	14 (21%)	19 (25%)	10 (20.0%)	15 19%)	0 (0%)	12 (16%)
**1**	30 (53%)	34 (48%)	24 (35%)	40 (52%)	26 (52.0%)	33 (41%)	25 (64%)	31 (43%)
**2**	15 (26%)	10 (14%)	17 (25%)	9 (12%)	8 (16.0%)	17 (21%)	8 (21%)	16 (22%)
**3**	12 (21%)	9 (13%)	13 (19%)	8 (10%)	6 (12.0%)	15 (19%)	6 (15%)	14 (19%)
**NAS (*n* (%))**								
**2**	1 (1.8%)	4 (6%)	1 (2%)	7 (9%)	0 (0.0%)	4 (5%)	0 (0%)	2 (3%)
**3**	10 (18%)	8 (11%)	7 (10%)	16 (21%)	9 (18%)	10 (12%)	7 (18%)	7 (10%)
**4**	12 (21%)	22 (31%)	15 (22%)	24 (31%)	13 (26%)	21 (26%)	10 (26%)	17 (23%)
**5**	19 (33%)	12 (17%)	16 (24%)	16 (21%)	10 (20%)	21 (26%)	10 (26%)	19 (26%)
**6**	12 (21%)	12 (17%)	16 (24%)	10 (13%)	14 (28%)	12 (15%)	10 (26%)	14 (19%)
**7**	3 (5%)	12 (17%)	12 (18%)	4 (5%)	4 (8%)	12 (15%)	2 (5%)	13 (18%)
**8**	0 (0%)	1 (1%)	1 (2%)	0 (0%)	0 (0%)	1 (1%)	0 (0%)	1 (1%)
**NASH (*n* (%))**	5 (9%)	11 (16%)	7 (10%)	20 (26%)	0 (0%)	14 (17%)	0 (0%)	0 (0%)

SD: standard deviation; BMI: body mass index.

**Table 3:** Classification trees.

Histologic Assessment	Classification Tree Using Mean ALT Only (96 weeks)	Classification Tree Using All Variables * (96 weeks)
Fibrosis	Mean ALT < 77.1 → Improvement Mean ALT ≥ 77.1 → Progression	Mean ALT < 77.1 → Improvement Mean ALT ≥ 77.1 → Progression
NAS	Mean ALT < 68.6 → Improvement Mean ALT ≥ 68.6 → Progression	Percent Change < −68.4% → Improvement Percent Change ≥ −68.4% → Progression
NASH	Mean ALT < 61.7 → Improvement Mean ALT ≥ 61.7 → Progression	ALT at week 96 < 56.5 → Improvement ALT at week 96 ≥ 56.5 → Progression
NASH + Fibrosis	Mean ALT < 65.2 → Improvement Mean ALT ≥ 65.2 → Progression	ALT at week 96 < 56.5 → Improvement ALT at week 96 ≥ 56.5 → Progression

* Age, gender, ethnicity, ALT cluster, ALT at baseline and at 96 weeks, % change in ALT from baseline, coefficient of variation of ALT, mean ALT.

**Table 4:** Classification and Regression Trees (CART), Model Performance with 5-fold cross validation across 20 replications—average (standard deviation).

	Fibrosis	NAS	NASH	NASH + Fibrosis
Mean ALT (96 weeks) *				
ROC	60.59 (13.31)	57.4 (10.1)	73.70 (14.25)	74.59 (14.22)
Sensitivity	55.65 (13.70)	52.3 (20.1)	65.79 (14.10)	66.21 (18.38)
Specificity	73.82 (10.77)	62.9 (17.9)	86.78 (11.87)	86.91 (11.98)
Fully adjusted Model (96 weeks) **				
ROC	58.56 (11.1)	63.0 (17.7)	81.84 (11.0)	77.78 (11.22)
Sensitivity	56.50 (15.12)	56.0 (17.5)	80.52 (15.27)	71.76 (21.59)
Specificity	64.63 (14.81)	74.7 (16.3)	82.99 (11.55)	80.81 (12.72)

* Mean ALT as predictor; ** age, gender, ethnicity, ALT cluster, ALT at baseline and at 96 weeks, % change in ALT from baseline, coefficient of variation of ALT, mean ALT.
